# How Large Are Gender Differences in Toy Preferences? A Systematic Review and Meta-Analysis of Toy Preference Research

**DOI:** 10.1007/s10508-019-01624-7

**Published:** 2020-01-27

**Authors:** Jac T. M. Davis, Melissa Hines

**Affiliations:** Gender Development Research Centre, University of Cambridge, Free School Lane, Cambridge, CB2 3RQ UK

**Keywords:** Gender, Children, Toys, Meta-analysis, Gender identity, Gender role

## Abstract

It is generally recognized that there are gender-related differences in children’s toy preferences. However, the magnitude of these differences has not been firmly established. Furthermore, not all studies of gender-related toy preferences find significant gender differences. These inconsistent findings could result from using different toys or methods to measure toy preferences or from studying children of different ages. Our systematic review and meta-analysis combined 113 effect sizes from 75 studies to estimate the magnitude of gender-related differences in toy preferences. We also assessed the impact of using different toys or methods to assess these differences, as well as the effect of age on gender-related toy preferences. Boys preferred boy-related toys more than girls did, and girls preferred girl-related toys more than boys did. These differences were large (*d* ≥ 1.60). Girls also preferred toys that researchers classified as neutral more than boys did (*d* = 0.29). Preferences for gender-typical over gender-atypical toys were also large and significant (*d* ≥ 1.20), and girls and boys showed gender-related differences of similar magnitude. When only dolls and vehicles were considered, within-sex differences were even larger and of comparable size for boys and girls. Researchers sometimes misclassified toys, perhaps contributing to an apparent gender difference in preference for neutral toys. Forced choice methods produced larger gender-related differences than other methods, and gender-related differences increased with age.

## Introduction

Gender-related toy preferences, and their origin and development, remain a controversial topic. Toys might influence children’s development of social and spatial skills (Jirout & Newcombe, 2015; Wong & Yeung, 2019) or signal later developmental changes such as sexuality (Li, Kung, & Hines, 2017) or aggressive behavior (Kung, Li, Golding, & Hines, 2018). Consequently, parents, educators, and policymakers want to know whether gendered toys might be influencing boys and girls differently (e.g., Bainbridge, 2018; Kamenetz & Turner, 2019; Tortorello, 2019). There are hundreds of scholarly articles documenting gender-related toy preferences, and these are often cited and shared in the popular press (e.g., Barford, 2014; Oksman, 2016). These articles, however, do not always agree on whether toys show gender differences and, for those that do, how large the differences are.

Anyone who has watched children play would probably conclude that girls and boys tend to prefer different toys, but researchers have not always been able to document these gender effects. Whereas some studies report large, stable effects for gender-related differences in children’s toy preferences (Alexander, Wilcox, & Woods, 2009; van de Beek, van Goozen, Buitelaar, & Cohen-Kettenis, 2009; Weinraub et al., 1984), others find ambiguous effects (Jacklin, Maccoby, & Dick, 1973), and still others find a mix of null and large effects (Campbell, Shirley, Heywood, & Crook, 2000; Serbin et al., 2001). Similarly, some find gender differences (i.e., different preferences in girls compared to boys), but not gender-specific preferences (i.e., a preference for same-sex over other sex toys), particularly in girls (e.g., Berenbaum & Hines, 1992), while others find both gender differences and gender-specific preferences, in both girls and boys (e.g., Pasterski et al., 2005). So, studies do not always find consistent gender effects on children’s toy preferences.

This apparent inconsistency may partly be due to variations in research design. Toy preference studies do not always use the same toys. The specific toys used in a study, and whether those toys are classified as boy-related toys, girl-related toys, or neutral toys, is not standardized across toy preference research. Additionally, toy preference studies do not always use the same methods for measuring preference. Preference can be measured in many ways, including assessing children’s actual play behavior, children’s visual attention, or children’s stated preferences, or itemizing the toys that children own or want to own. Finally, the results of toy preference studies may have changed over time, with more recent studies finding different results to earlier studies. Any of these variations may influence the size of the gender effect and may partly explain why toy preference studies do not always produce the same results.

When comparing the findings of different toy preference studies (e.g., over time), an underlying assumption is that the studies’ measurement methods should produce comparable results. Alternatively, discrepancies in the results of individual studies are often thought to result from differences in the studies’ methods. These assumptions can be tested empirically using meta-analytic techniques. Individual studies typically use a single method to measure toy preferences, so meta-analytic comparisons across studies provide a way to determine whether, and how, study methods might affect results. Similarly, meta-analytic techniques can be used to examine the sizes of gender-related differences for specific individual toys and to examine the effect of factors such as age or the dates of studies on research results. The following sections review prior research on children’s gender-related toy preferences, focusing on the potential for meta-analytic techniques to help explain the sometimes conflicting findings in this area of research.

Studies on gender-related toy preferences do not always agree on terminology, so the present review defines some key terms as follows. We refer to the set of toys that researchers think are stereotyped as for boys, or that they think boys will prefer, as *boy*-*related* toys, and we refer to the set of toys that researchers think are stereotyped for girls, or that they think girls will prefer, as *girl*-*related* toys. Together, these boy-related toys and girl-related toys are referred to as *gender*-*related* toys. We use *gender differences* to refer to average differences between boys and girls. An example of a gender difference might be the difference between boys’ preference for a doll or girls’ preference for a doll. Similarly, we use *gender*-*specific preferences* to refer to average differences between boy-related toys and girl-related toys. An example of a gender-specific preference might be the difference between boys’ preference for a doll and boys’ preference for a vehicle. Together, these gender differences and gender-specific preferences are referred to as *gender effects*.

### Gender-Related Toys

Studies of children’s gender-related toy preferences do not always use the same toys, and researchers do not always select toys in a systematic way. Sometimes, researchers select and categorize toys based on the toys’ gender stereotypes, as previously rated by adults (e.g., Idle, Wood, & Desmarais, 1993; Le Maner-Idrissi, 1996; Zucker, 1977). Similarly, researchers sometimes select toys for a study and then ask adults to rate their gender stereotyping or gender appropriateness (e.g., Gugula, 1999; Guinn, 1984). Another approach is to cite previous work as the basis for selecting and categorizing toys, though researchers do not always indicate whether the current study was a direct replication or included some variation on the toy set (e.g., Karpoe & Olney, 1983). Alternatively, some investigators attempt to infer a consensus from previous work and choose toys that they judge to have been consistently gender-related (e.g., Lloyd & Smith, 1985). Finally, some researchers do not rely on predetermined sets of toys, but instead observe girls and boys playing in natural settings. To sort toys into gender categories, researchers using this approach may subsequently ask adults to rate the gender typicality of the toys (e.g., Downs, 1983), or they may group the toys by some other features that they assume are gender-typed, for example, toys that are used for art or for construction (Nelson, 2005).

Researchers can also be inconsistent about describing potentially relevant characteristics of the toys selected for study. For instance, some researchers have investigated the impact of color on children’s gender-related toy preference (e.g., Jadva, Hines, & Golombok, 2010; Weisgram, Fulcher, & Dinella, 2014; Wong & Hines, 2015), but many researchers do not report the color of the toys used in their studies. Other characteristics, such as shape, tactile softness, or newness of the toys, or the toys’ utility for social role play, mechanical movement, or propulsion, may also be important in determining children’s gender-related toy preferences (Benenson, Liroff, Pascal, & Cioppa, 1997; Escudero, Robbins, & Johnson, 2013; Hassett, Siebert, & Wallen, 2008; Jacklin et al., 1973; Jadva et al., 2010; Lobel & Menashri, 1993; Zosuls et al., 2009), but few studies have reported these features for the toys used in their research. Finally, researchers usually do not report statistical information needed to calculate effect sizes for individual toys, but instead report statistical results only for broader toy groupings.

### Methods of Measuring Toy Preferences

Gender-related toy preference is a broad category, and we focus here on direct measurements of children’s gender-related toy preferences. We consider direct measurements to include any measurements based on children’s self-reported preferences or on children’s behavior, and we do not include measurements based on reports from parents, teachers, or retrospectively from adult participants. Direct measurements can differ from one study to another, but they can be grouped into four general categories: free play, visual preference, forced choice, and naturalistic approaches. Some variation exists among studies within each of these categories, but they are more similar to one another than they are to studies in the other categories. While all free play studies, for example, are not exactly the same, they are more similar to each other than they are to visual preference, forced choice, or naturalistic studies. In this section, we describe the defining characteristics of each method, with examples.

#### Free Play

In free play studies, children are presented with a set of toys and allowed to play with them in an unstructured way. Toys are selected by the experimenter or other adults, and researchers sort the toys into gender categories. Sometimes additional toys are included that have been assigned an a priori gender-neutral status as well. The measure of interest is typically the amount or proportion of time that children spend playing with each toy or group of toys. Free play studies are primarily carried out in laboratory settings, but may also be conducted in schools or homes. The defining characteristic of free play studies is that children’s preference is measured based on their play behavior, but that the starting set of toys is determined by someone other than the child.

One common formulation of a free play study is to bring a child into a prepared room containing a set of toys and then to give the child a set amount of time to play with the toys. For example, a study by Serbin, Connor, Burchardt, and Citron (1979) placed children in a small room with a row of six toys and allowed children to play for 3 min. The six toys were selected by the experimenters as being stereotypically appealing to boys (three toys) or to girls (three toys). A similar formulation of the free play paradigm has been used by many subsequent studies, with minor variations. For example, Pasterski et al. (2005) used a similar procedure. However, this later study used more toys and different toys, placed the toys in a circle around the child instead of in a row, included a set of neutral toys as well as girl- and boy-related toys, and allowed each child to play for 8 min instead of three. In studies like these, children may play with more than one toy at once, or with no toys at all, resulting in a wider range of results than may be available when children are forced to choose one option from a set. Constraints on the child’s play are still present in the form of a limited set of available toys and a limited time available for play.

Other studies using a free play approach have observed children over a longer time and have assessed a wider range of behaviors, although the set of behaviors is still determined by adults. A common approach is to observe children at school or preschool and compare their play activities using a predetermined checklist. An early example of this approach was Fagot and Patterson’s (1969) study of gender-typed behavior. Researchers observed each child for a 10–15-s interval about once every 5 min across 70 min of free play. Children’s behavior in each interval was coded according to a checklist of 28 responses that had been previously defined by the researchers. The checklist included gender-related activities, such as play with girl-related and boy-related toys, as well as neutral and non-play responses, such as talking to a teacher. More recently, a similar approach has been used by Martin et al. (2013) in an investigation of the role of peers in children’s gender-typed play.

#### Visual Preference

In visual preference paradigms, children are presented with toys or with images of toys, either sequentially or side-by-side. Researchers using this paradigm select the toys or images to be used and assign them an a priori status as boy-related, girl-related, or neutral. The length of time that children look at a toy is scored by hand or with the help of cameras or eye-tracking software. The measure of interest is typically the proportion of time spent looking at each toy or category of toy, usually as a proportion of the overall time the child was attentive. The defining characteristic of visual preference paradigms is that children’s preferences were measured based on visual attention, rather than on physical contact or explicit choice.

Visual preference studies usually present children with images of toys, rather than the actual items. For example, in a study by Escudero, Robbins, and Johnston (2013), infants were placed on a caregiver’s lap and presented with two side-by-side images of a face and a vehicle, using multiple trials varying the faces (a real face and a doll face) and the vehicles (a real car and a toy car). Infants’ preferences were measured using a corneal reflection eye tracker. Similarly, Jadva, Hines, and Golombok (2010) presented infants with a series of side-by-side line drawings of dolls and vehicles, varying the color and left/right placement of the stimuli. Infants’ faces were recorded on video and later scored for gaze direction.

#### Forced Choice

In forced choice studies, the experimenter presents children with a series of choices, usually between two toy options, one of which is boy-related, and the other of which is girl-related. The choices are typically presented as a series of questions with picture aids, and the measure of interest is the proportion of choices that are gender-related in each direction out of the total number of trials. The exact implementation may vary, but the key features of forced choice methods are restricted options and, usually, a requirement to choose in front of the experimenter.

Forced choice methods have been used in toy preference research for decades. For example, DeLucia (1963) used black and white photographs of 24 toys, balanced for size, monetary value, and intricacy of movable parts. Toys were categorized as girl-related or boy-related, based on the rankings of adults regarding their appeal to boys and girls. Children were presented with pairs of pictures, asked to choose which of the pair they preferred, and given a score based on the number of the same gender-related choices that they made. Alexander and Hines (1994) used a series of cards to measure children’s gender-related interests, including toy preferences. In the toy preference portion of their assessment, each card included two scenes of stick figures engaging in play with different toys that the researchers had classified as girl-related or boy-related. The child was asked to choose his or her preferred option from each card, and given a score based on their same gender-related choices.

#### Naturalistic Methods

Naturalistic studies are designed to reduce the influence of the experimenter on the stimuli available and on the behavior of those being observed. These methods attempt to measure preferences without any a priori determination of the toys that are available for children to choose. Some naturalistic studies measure the gender-related toys that children own. For example, Nelson (2005) inventoried children’s toy collections in their homes and sorted the toys that children owned into gender-related categories. However, inventory studies are sometimes criticized because these toys were purchased for children by adults, so a child’s toy collection may reflect the preferences of adult purchasers, as well as the preferences of the child. Therefore, other studies have attempted to overcome this limitation by measuring children’s requests for toys, rather than the toys that they actually own. For example, Downs (1983) collected children’s letters to Santa and measured the number of gender-related toys that children had requested as Christmas presents. The measure of interest varies more in naturalistic than in other types of studies, but typically the proportion of boys and girls owning or requesting each toy or category of toy is reported. Naturalistic studies represent the only widely used approach where researchers or other adults do not make a priori decisions, independent of children, as to which toys are available to be preferred, or are of interest.

### Child Age

Gender-related differences in children’s toy preferences might change with age. Based on their early gender-related toy interests, children might gravitate to different social environments, enhancing their early preferences and producing a linear increase in gender-related differences with age (e.g., Golombok et al., 2008). Alternatively, children might be expected initially to adopt more consistent gender-related behaviors as they develop an understanding of their own gender (Kohlberg, 1966), but then to become more flexible in later years, as they begin to understand that social conventions are culturally determined and changeable (Carter & Patterson, 1982). Thus, gender effects might increase with age, or they might show a curvilinear effect with an initial increase, followed by a later decrease, in gender-related differences.

### Year of Study

Changes in the wider social and political context may have affected toy preference research over time. Children’s toy preferences have been studied over more than five decades, since at least the 1960s (DeLucia, 1963). During this time, some meta-analytic findings have suggested that gender differences in some areas have decreased, for example, in some aspects of cognitive performance (Feingold, 1988). Not all reviews find a decrease in gender differences, however. For example, a meta-analysis of 50 years of data found that the gender difference in body image had increased over time (Feingold & Mazzella, 1998). Across a similar time period, academic and wider social perspectives on gender and toys may have changed, and these changes may have affected the results of toy preference studies.

Additionally, the perceived value of children’s gender-related behavior has changed over time. In early research, gender-related behavior was seen as necessary to healthy development, and researchers sought to identify conditions that would encourage children to engage in behaviors that were “sex-appropriate,” and to document the consequences of behaviors that were not (e.g., Anastasiow, 1965; Barkley, Ullman, Otto, & Brecht, 1977). Subsequently, however, academic approaches shifted, to view gender-related behavior as incidental (e.g., Maccoby, 1990) and, in some cases, harmful (e.g., Gunderson, Ramirez, Levine, & Beilock, 2012) to healthy development. This shift in research perspective raises the question of whether there were corresponding changes in study results over time.

### Previous Reviews of Toy Preference Research

Previous reviews of toy preference research have typically been narrative reviews. One meta-analysis has been conducted on a subset of toy preference studies using free play methods (Todd et al., 2018). The present meta-analysis extended this previous effort by including, and comparing, different methods for measuring toy preferences. Additionally, the present meta-analysis included effect sizes for gender-specific effects (e.g., how much boys prefer boy-related toys to girl-related toys), while the previous meta-analysis focused on gender differences. Further, the present meta-analysis examined whether gender differences in toy preferences were smaller or larger for specific types of toys (dolls and vehicles), while the previous meta-analysis focused only on broader groups of gender-related toys.

### The Current Review and Meta-Analysis

Here we conduct a systematic review and meta-analysis of gender-related effects on children’s toy preferences. The present review sought to establish: (1) the magnitude of gender-related effects on children’s toy preferences; (2) whether specific toys (dolls and vehicles) were more or less gender-related than broader toy groupings; (3) whether different methods of measuring preference (free play, visual preference, forced choice, or naturalistic) found different gender effects; (4) whether child age was related to the magnitude of gender effects on children’s toy preferences; and (5) whether year of study publication was related to the magnitude of gender effects on children’s toy preferences. To assess confidence in the meta-analysis results, we also include a set of tests for publication bias, including funnel plots and regression tests.

## Method

### Systematic Search Method

We located studies through an online search of journal indexing databases (Scopus, ScienceDirect, ProQuest, and EBSCO), dissertation abstracts, and Google Scholar. We discontinued our literature search in March 2014. The systematic search was conducted in English-indexed journals. If the paper provided an English-language abstract and was judged eligible for inclusion, it was translated.

Search keywords included terms relevant to the predictor (gender), the outcome (toy preference), and the population (children). Each search query therefore contained three elements, including synonyms and more specific terms for each (e.g., “gender” or “sex” or “male” or “boy” and “play” or “toy” or “preference” and “children”). These terms were combined using Boolean operators to take advantage of the functionality of each database. We also recognized that the terms used for gender-related toy preference may have changed over time, and so searched specific names of toy preference measures referenced in a book of gender tests (Beere, 1990), and the reference lists of included studies.

### Inclusion Criteria

We designed inclusion criteria that would retain a large sample of effects while limiting the analysis to studies that were statistically comparable. Studies were included if they provided empirical data on toy preferences in children aged 11 years or younger. Studies must have included gender in the report as an explanatory variable, but the study did not have to be explicitly or solely focused on gender differences. Studies must also have reported toy preferences as outcome measures. Toy preferences had to be obtained from children directly; studies that measured toy preferences through parent report, or through retrospective reporting from adult participants, were not eligible for inclusion in the present review.

We included studies with any of the following research designs: non-randomized designs, comparing boys and girls on one or more measures of toy preference; randomized or non-randomized designs testing another predictor of toy preferences, but including in the results a breakdown of the outcome measure by participant sex; and longitudinal designs, testing changes in gender-related toy preferences over time, with results presented along with some report of how results differed by participant sex. Only data from typically developing children were included in the meta-analyses; data from participants that were selected on the basis of their gender non-conformity, or a medical diagnosis, were not included.

### Analyses

#### Effect Size Calculation

Each study had corresponding effect sizes calculated and converted for the meta-analysis, using standard procedures (Borenstein, Hedges, Higgins, & Rothstein, 2011; Lipsey & Wilson, 2001). For the primary meta-analyses, up to five effect sizes (standardized mean differences) were calculated for each study: (1) gender difference in preference for boy-related toys; (2) gender difference in preference for girl-related toys; (3) boys’ gender-specific preference for boy-related over girl-related toys; (4) girls’ gender-specific preference for girl-related over boy-related toys; and (5) gender difference in preference for neutral toys. Effect sizes were calculated so that if the effect was positive, it was in the direction that would be expected a priori; for example, if girls prefer girl-related over boy-related toys, the effect is positive; if boys prefer boy-related over girl-related toys, the effect is positive. For neutral toys, a positive effect size would indicate that boys preferred the toys more than girls did. Studies could contribute effect sizes to all five meta-analyses, so these meta-analyses were not independent.

Study statistics were collected and transformed in the following order of preference: means and SD; direct reporting of effect sizes (e.g., standardized mean difference, correlation coefficient, odds ratio); effect measures with magnitude and direction (e.g., regression coefficients and SE, mean differences); raw numbers; results of test statistics (e.g., *t* values, *p* values); or digitized numbers read from figures using a web-based plot digitizer program (WebPlotDigitizer version 3.9; Rohatgi, 2015).

#### Meta-analysis Models

We used multilevel meta-analysis models to properly account for correlated data structures within studies that reported on several groups at once (for example, studies that used a longitudinal design, with children measured at multiple ages, or papers reporting on multiple groups). Meta-analysis models used inverse variance weights and restricted maximum likelihood estimation.

We also ran sensitivity analyses to test that the results were robust to using the following: standard random-effects meta-analysis, multilevel meta-analysis, and multivariate parameterization of the multilevel meta-analysis. Substantive results were consistent across all types of analysis.

#### Types of Toys: Dolls and Vehicles

Four multilevel meta-analyses examined effect sizes for gender-related preferences for two specific categories of toys: dolls and vehicles. These assessed the gender difference in preference for dolls, the gender difference in preference for vehicles, boys’ gender-specific preference for vehicles, and girls’ gender-specific preference for dolls. We statistically compared the results of the meta-analyses of dolls and vehicles to the results of the meta-analyses of the broader toy groupings, using a modified *t* test for comparing standardized effect sizes. We could not include similar analyses for toy categories other than dolls and vehicles, because insufficient numbers of studies reported results for any other specific toy categories.

#### Study Method, Child Age, and Year of Publication

We used moderator analyses to test for the effects of the study method (free play, visual preference, forced choice, and naturalistic), child age, and year of publication. Studies were not excluded from the main meta-analyses if they did not report information on a moderator (e.g., if child age was unclear), but they were excluded from the analysis for that moderator. In addition, all studies were included in the analysis of method as a moderator, as all the studies fit into one of the four categories of methods. We used multivariate multilevel mixed effects meta-regression models.

#### Publication Bias

A series of funnel plots and corresponding regression tests (Egger, Smith, Schneider, & Minder, 1997) assessed whether the effect sizes differed for small and large studies. Since large studies tend to be published even if they report small effect sizes, a high rate of large studies with small effect sizes and small studies with large effect sizes would suggest publication bias in the sample.

#### Statistical Software

All analyses were conducted using the statistical software *R*. Specific packages included metafor for frequentist meta-analysis models (Viechtbauer, 2010) and basic plot functions to create figures.

## Results

### Systematic Search Results

**Fig. 1:**
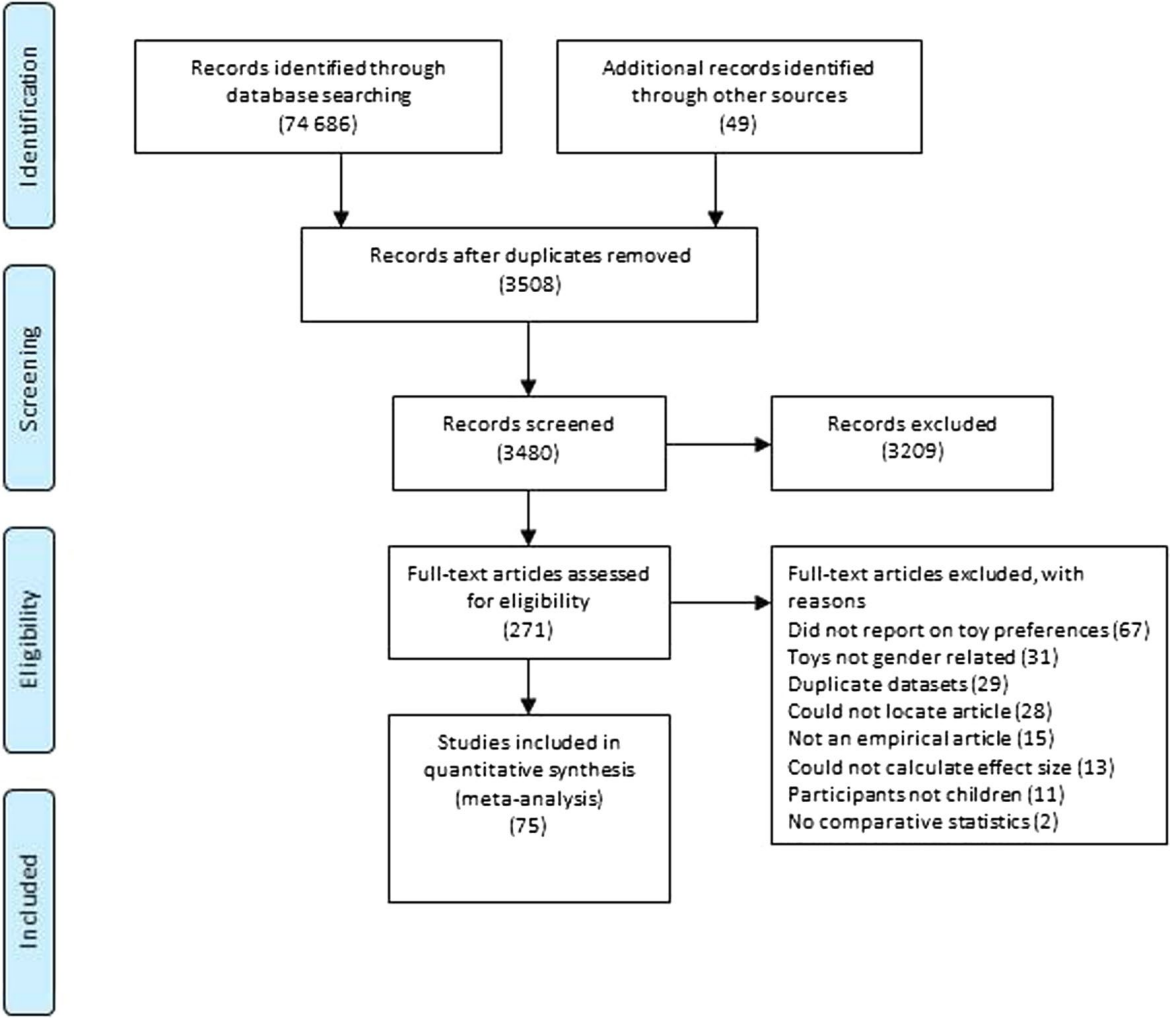
PRISMA flow diagram for attrition of publications included in the systematic review. Numbers in brackets are number of sources

The systematic search identified 3,508 unique sources. Twenty-eight sources (0.8%) could not be obtained to review for eligibility (these were: 1 erratum; 1 reply; 3 sources in non-English-language journals that we could not access; 12 sources in English-language journals that we could not access, all published between 1973 and 1987; 1 source in a non-indexed journal; 7 dissertations; 1 conference paper; and 2 sources with no reference information). Of the 3,508 sources obtained, 981 were marked provisionally eligible according to the title and keywords, and, on inspection of the abstract, 271 of these were marked provisionally eligible. Of these, on inspection of the full text, 196 studies had no comparative data, did not report on toy preferences, did not include children as participants, or did not report sufficient statistics to calculate an effect size. These studies were excluded, as reported in the Preferred Reporting Items for Systematic Reviews and Meta-Analyses (PRISMA) flow diagram (Moher, Liberati, Tetzlaff, Altman, & The PRISMA Group, 2009) in Fig. 1.^1^ The final set of 75 papers eligible for the meta-analysis contained 113 effect sizes for gender-related differences in toy preferences. The number of effect sizes exceeded the number of papers because some studies contained multiple effect sizes (e.g., because of multiple age groups within a study or multiple studies within a paper).

### Description of Toy Preference Studies

Study characteristics are summarized in Table 1.

**Table 1:** List of studies included in the meta-analysis of gender-related differences in toy preferences, with key characteristics

Study	Subgroup	Country	Age (years)	Measure	*n* boys	*n* girls
Alexander and Hines (1994)^a^		USA	4.00	Forced choice	28	32
Alexander, Wilcox, and Woods (2009)^a^		USA	0.50	Visual preference	17	13
Alexander, Wilcox, and Farmer (2009)^a^		USA	0.33	Visual preference	21	20
Alexander and Saenz (2012)^a^		USA	1.58	Free play	47	37
Anastasiow (1965)^a^		USA	5.50	Forced choice	60	60
Ashton (1983)^a^		USA	4.00	Multiple	16	16
Banerjee and Lintern (2000)^a^	4-year-olds	UK	5.33	Forced choice	11	10
	6-year-olds	UK	6.42	Forced choice	10	11
	8-year-olds	UK	9.08	Forced choice	10	12
Barkley et al. (1977)^a^		USA	7.33	Free play	40	40
Benenson et al. (1997)^a^		Canada	4.58	Free play	18	16
Berenbaum and Hines (1992)^a^		USA	5.42	Free play	18	15
Berenbaum and Snyder (1995)^a^		USA	7.50	Free play	19	13
Blakemore, LaRue, and Olejnik (1979)^a^	2-year-olds	USA	2.00	Forced choice	10	10
	4-year-olds	USA	4.00	Forced choice	10	10
	6-year-olds	USA	6.00	Forced choice	10	10
Boldizar (1991)^a^		USA	10.75	Forced choice	74	71
Bradbard and Parkman (1984)^a^		USA	4.00	Naturalistic	99	102
Caldera, Huston, and O’Brien (1989)^a^		USA	1.67	Free play	40	40
Campbell et al. (2000)^a^	18-month-olds	UK	1.75	Visual preference	29	19
	3-month-olds	UK	0.25	Visual preference	29	19
	9-month-olds	UK	0.75	Visual preference	29	19
Cherney et al. (2003)^a^		USA	2.50	Free play	15	15
Cherney and Dempsey (2010)^a^		USA	4.25	Free play	19	12
Corter and Jamieson (1977)^a^		Canada	1.25	Free play	10	10
DeLucia (1963)^a^	First grade	USA	6.58	Forced choice	23	23
	Second grade	USA	8.25	Forced choice	10	10
	Third grade	USA	9.17	Forced choice	10	10
	Fourth grade	USA	10.00	Forced choice	10	10
	Kindergarten set A	USA	5.83	Forced choice	15	15
	Kindergarten set B	USA	5.83	Forced choice	45	45
Doering et al. (1989)^a^		Canada	7.25	Free play	15	15
Downs (1983)^a^		USA	7.25	Naturalistic	77	77
Eisenberg, Tryon, and Cameron (1984)^a^		USA	4.58	Free play	26	25
Escudero, Robbins, and Johnson (2013)^a^	Experiment 1A	Australia	0.25	Visual preference	12	12
	Experiment 1B	Australia	0.25	Visual preference	12	12
Fagot and Patterson (1969)^a^		USA	3.42	Free play	18	18
Fagot and Leinbach (1989)^a^	Early labeler	USA	1.50	Forced choice	11	12
	Late labeler	USA	1.50	Forced choice	11	14
	Early labeler	USA	2.25	Forced choice	11	12
	Late labeler	USA	2.25	Forced choice	11	14
Fein, Johnson, Kosson, Stork, and Wasserman (1975)^a^		USA	1.67	Free play	11	13
Fisher-Thompson and Burke (1998)^a^		USA	9.08	Forced choice	60	60
Frasher, Nurss, and Brogan (1980)^a^		USA	5.58	Forced choice	55	55
Freeman (1995)^a^		USA	7.92	Naturalistic	354	470
Fridell, Owen-Anderson, Johnson, Bradley, and Zucker (2006)^a^		Canada	6.77	Forced choice	96	38
Goble et al. (2012)^a^		USA	4.33	Free play	133	131
Goldman, Smith, and DuWayne Keller (1982)^a^		USA	1.50	Free play	31	26
Gugula (1999)^a^		Canada	3.75	Free play	24	24
Guinn (1984)^a^		USA		Forced choice	66	69
Henderson and Berenbaum (1997)^a^	Girls with boy co-twin	USA	5.08	Free play	0	35
	Girls with girl co-twin	USA	5.50	Free play	0	36
	Girls with brother	USA	5.25	Free play	0	20
Idle, Wood, and Desmarais (1993)^a^		Canada	3.83	Free play	10	10
Jacklin, Maccoby, and Dick (1973)^a^	Experiment 1	USA	1.08	Free play	20	20
	Experiment 2	USA	1.08	Free play	20	20
Jadva, Hines, and Golombok (2010)^a^	12-month-olds	UK	1.08	Visual preference	20	20
	18-month-olds	UK	1.67	Visual preference	20	20
	24-month-olds	UK	2.17	Visual preference	20	20
Karpoe and Olney (1983)^a^		USA	10.75	Free play	15	15
Lamminmäki et al. (2012)^a^		Finland	1.17	Free play	21	26
Le Maner-Idrissi (1996)^b^		France	1.83	Free play	24	24
Lloyd and Smith (1985)^a^		UK	1.83	Free play	15	15
Martin et al. (2013)^a^	Wave 1	USA	4.25	Free play	156	136
	Wave 2	USA	4.25	Free play	156	136
	Wave 3	USA	4.25	Free play	156	136
	Wave 4	USA	4.25	Free play	156	136
McHale et al. (2004)^b^		USA	10.83	Self-report	97	103
Meyer-Bahlburg et al. (2004)^a^		USA	8.5	Free play	16	25
Moller and Serbin (1996)^a^		Canada	2.92	Free play	28	29
Nelson (2005)^a^		Sweden	4.00	Naturalistic	77	75
Nordenström et al. (2002)^a^		Sweden	5.25	Free play	0	31
O’Brien, Huston, and Risley (1983)^b^		USA	2.00	Free play	24	17
O’Brien and Huston (1985)^b^		USA	1.58	Free play	24	28
Pasterski et al. (2005)^a^		USA and UK	6.75	Free play	25	27
Pasterski et al. (2011)^a^		USA and UK		Forced choice	17	26
Peretti and Sydney (1986)^a^		USA	2.50	Free play	75	75
Powlishta et al. (1993)^a^		Canada	2.92	Free play	28	29
Raag (1999)^a^		USA	4.67	Free play	57	50
Rekers and Yates (1976)^a^		USA	5.50	Free play	60	60
Richardson and Simpson (1982)^a^		USA		Naturalistic	359	391
Robinson and Morris (1986)^a^	36-month-olds	USA	3.00	Naturalistic	46	43
	48-month-olds	USA	4.00	Naturalistic	46	43
	60-month-olds	USA	5.00	Naturalistic	46	43
Rodgers, Fagot, and Winebarger (1998)^a^		USA	8.25	Free play	86	80
Roopnarine (1986)^a^	10-month-olds	USA	0.83	Free play	4	5
	14-month-olds	USA	1.17	Free play	5	9
	18-month-olds	USA	1.50	Free play	5	6
Rotsztein and Zelazo (2000)^b^	13-month-olds	Canada	1.08	Free play	14	14
	22-month-olds	Canada	1.83	Free play	14	14
	31-month-olds	Canada	2.58	Free play	14	14
Schau, Kahn, Diepold, and Cherry (1980)^a^		USA	4.00	Free play	26	26
Seegmiller, Suter, Dunivant, and Baldemor (1979)^b^	Test 1	USA	4.00	Forced choice	99	86
	Test 2	USA	4.00	Forced choice	100	113
Serbin et al. (1979)^b^		Canada	4.25	Free play	36	26
Serbin et al. (2001)^a^	12-month-olds	Canada	1.00	Visual preference	8	12
	18-month-olds	Canada	1.50	Visual preference	15	15
	23-month-olds	Canada	1.92	Visual preference	14	13
Servin, Bohlin, and Berlin (1999)^a^	1-year-olds	Sweden	1.00	Free play	19	19
	3-year-olds	Sweden	3.00	Free play	13	18
	5-year-olds	Sweden	5.00	Free play	14	21
Servin, Nordenström, Larsson, and Bohlin (2003)^a^		Sweden	5.75	Forced choice	0	26
Stagnitti, Rodger, and Clarke (1997)^a^		Australia	5.00	Free play	18	18
Turner and Gervai (1995)^a^	Budapest	Hungary	4.25	Forced choice	33	31
	Cambridge	UK	4.17	Forced choice	26	30
van de Beek et al. (2009)^b^		The Netherlands	1.17	Free play	63	63
Wilansky-Traynor and Lobel (2008)^b^	Sample 1	Canada	5.50	Free play	27	30
	Sample 2	Canada	5.50	Free play	30	29
Wong (2012)^a^	Time 1	UK	2.33	Free play	56	70
	Time 2	UK	2.92	Free play	56	70
Wood, Desmarais, and Gugula (2002)^a^		Canada	3.92	Free play	24	24
Zosuls (2009)^b^	17-month-olds	USA	1.42	Free play	36	46
	21-month-olds	USA	1.75	Free play	36	46

^a^Study location was reported in the paper

^b^Study location was inferred from the location of the primary author’s affiliation

The average age of children in toy preference studies ranged from a minimum of 3 months (Alexander, Wilcox, & Farmer, 2009; Campbell et al., 2000; Escudero et al., 2013) to a maximum of 11 years (Boldizar, 1991; McHale, Kim, Whiteman, & Crouter, 2004). The number of studies published on gender-related toy preferences rose in the late 1970s to early 1980s, and new studies continued to be published throughout the 1990s, 2000s, and to the present. Most studies were conducted in the U.S., Canada, and the UK, but studies were also conducted in Australia, Finland, Sweden, and Israel (see Table 1).

**Fig. 2:**
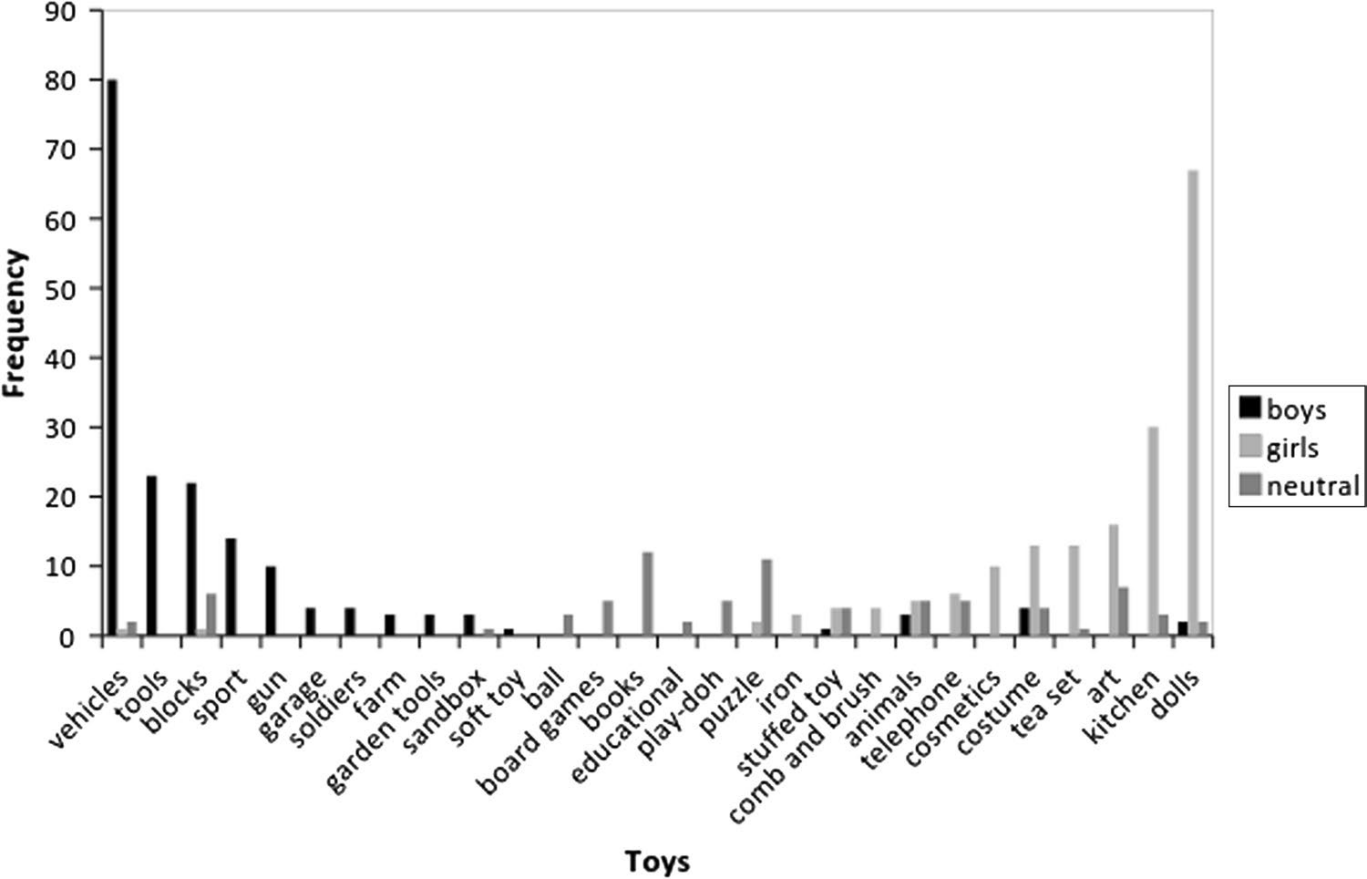
Toys used as girl-related, boy-related, and neutral toys as listed in method sections of studies included in the meta-analysis. Studies could contribute more than one toy to the figure. These toys were mentioned in method sections of studies, but data were not typically reported for each individual toy. Most studies reported statistics for groups of toys, but not for individual toys

Studies’ operational definitions of boy-related toys, girl-related toys, and neutral toys were not always consistent and in some cases overlapped. Vehicles and guns were almost always categorized as boy-related. Dolls were almost always categorized as girl-related. Other types of toys included active toys, such as sandpits and skipping ropes; appearance-related toys, such as brush and comb sets and makeup kits; toys for arts and crafts activities, such as Play-Doh or clay; household-related toys, such as tea sets and toy stoves; structures, such as houses, parking garages, and castles; writing tools; musical instruments; as well as a range of other toys. Figure 2 shows the number of studies that used specific toys, and their author-defined gender-related classifications.

Dolls and vehicles, for girls and for boys, respectively, were frequently used in toy preference studies. Furthermore, when a study included only a single toy for each gender, it often included a doll as a girl-related toy and a vehicle as a boy-related toy. Therefore, there was enough information available about dolls and vehicles, specifically, to test whether these toys showed the same gender differences as broader groups of gender-related toys.

We therefore conducted two sets of analyses. First, we analyzed gender effects on children’s preferences for boy-related toys compared to girl-related toys, broadly defined by study authors. Second, we analyzed gender effects on children’s preferences for dolls compared to vehicles. We compared the results for the broader toy groupings to the results for dolls and vehicles, to check whether the broader results were replicated with only the smaller subset of well-defined and often used toys.

### Gender Effects on Toy Preferences

#### Gender Difference in Preference for Boy-Related Toys

The multilevel meta-analysis of the gender difference in preference for boy-related toys included a total of 108 effect sizes. Boys preferred boy-related toys more than girls did, and this effect was large and statistically significant (*d* = 1.83, 95% CI = 0.96–2.71, *p* < .001). The regression test for funnel plot asymmetry showed no evidence of publication bias, *t*(106) = − 0.49, *p *= .626.

#### Gender Difference in Preference for Girl-Related Toys

The multilevel meta-analysis of the gender difference in preference for girl-related toys included a total of 108 effect sizes. Girls preferred girl-related toys more than boys did, and this effect was large and statistically significant (*d* = 1.60, 95% CI = 0.76–2.43, *p* < .001), and not significantly different from the gender difference in preference for boy-related toys, *z* = 0.43, *p* = .665. The regression test for funnel plot asymmetry showed no evidence of publication bias, *t*(106) = − 1.29, *p *= .201.

#### Boys’ Gender-Specific Preference for Boy-Related Toys Over Girl-Related Toys

The multilevel meta-analysis of boys’ gender-specific preference for boy-related toys included a total of 104 effect sizes. Boys preferred boy-related toys to girl-related toys, and this effect was large and statistically significant (*d* = 3.48, 95% CI = 1.17–5.79, *p* = .003). The regression test for funnel plot asymmetry showed no evidence of publication bias, *t*(102) = − 1.37, *p *= .174.

#### Girls’ Gender-Specific Preference for Girl-Related Toys Over Boy-Related Toys

The multilevel meta-analysis of girls’ gender-specific preference for girl-related toys included a total of 109 effect sizes. Girls preferred girl-related toys to boy-related toys, and this effect was large and statistically significant (*d* = 1.21, 95% CI = 0.61–1.82, *p* < .001) and was not significantly different than boys’ gender-specific preference for boy-related toys over girl-related toys, *z* = 1.84, *p* = .066. The regression test for funnel plot asymmetry showed no evidence of publication bias, *t*(107) = − 1.35, *p *= .180.

#### Gender Difference in Preference for Neutral Toys

The multilevel meta-analysis of the gender difference in preference for neutral toys included a total of 27 effect sizes. Girls preferred neutral toys more than boys did, and this effect was small but significant (*d* = − 0.29, 95% CI = − 0.56 to − 0.02, *p* = .039). The regression test for funnel plot asymmetry suggested possible publication bias, *t*(25) = − 2.05, *p *= .051. A follow-up trim-and-fill analysis (Duval & Tweedie, 2000) estimated two missing studies on the left side of the funnel plot. The revised meta-analysis estimate still showed girls preferring neutral toys significantly more than boys did (*d* = − 0.29, 95% CI = − 0.55 to − 0.03, *p* = .029).

### Vehicles and Dolls Compared to Broader Gender-Related Groups of Toys

#### Gender Difference in Preference for Vehicles

The multilevel meta-analysis of the gender difference in preference for vehicles included a total of 28 effect sizes. Boys preferred vehicles more than girls did, and this effect was large and statistically significant (*d* = 2.44, 95% CI = 0.52–4.35, *p* = .013).

#### Gender Difference in Preference for Dolls

The multilevel meta-analysis of the gender difference in preference for dolls included a total of 29 effect sizes. Girls preferred dolls more than boys did, and this effect was large and statistically significant (*d* = 4.12, 95% CI = 0.22–8.03, *p* = .038) and significantly larger than the gender difference in preference for toy vehicles, *t*(55) = 4.04, *p* < .001.

#### Boys’ Gender-Specific Preference for Vehicles Over Dolls

The multilevel meta-analysis of boys’ gender-specific preference for vehicles included a total of 27 effect sizes. Boys preferred vehicles to dolls, and this effect was large and statistically significant (*d* = 3.10, 95% CI = 0.73–5.47, *p* = .010).

#### Girls’ Gender-Specific Preference for Dolls Over Vehicles

The multilevel meta-analysis of girls’ gender-specific preference for dolls included a total of 27 effect sizes. Girls preferred dolls to vehicles, and this effect was large but not statistically significant with a two-tailed test (*d* = 3.51, 95% CI = − 0.62 to 7.65, *p* = .095). It also was not significantly different from the effect size for boys’ preference for vehicles over dolls, *t*(52) = 0.87, *p* = .388.

#### Vehicles and Dolls Compared to Broader Toy Groupings

**Fig. 3:**
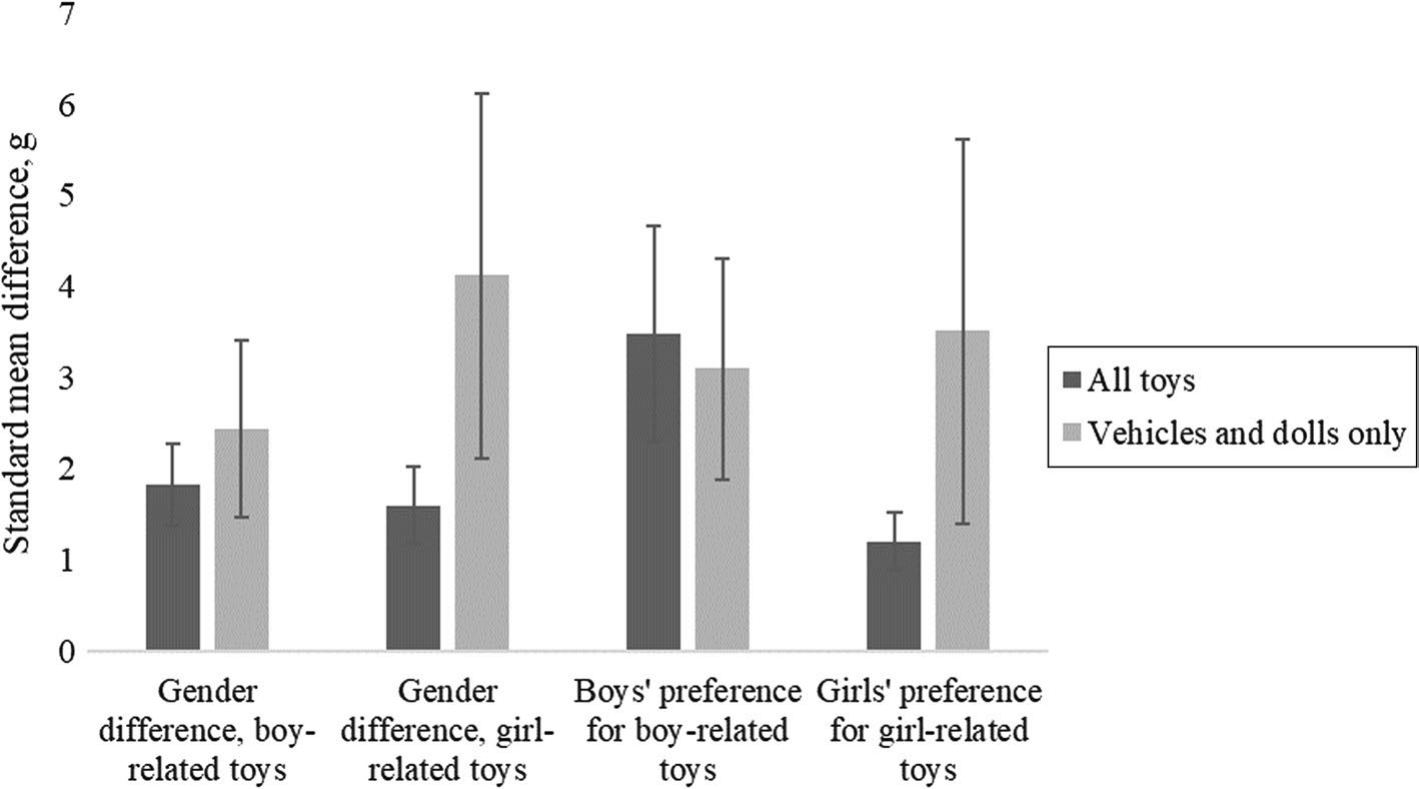
Standardized effect sizes for gender differences in children’s preferences for vehicles and dolls only compared to broad groups of boy-related, girl-related toys. Error bars show standard errors

Figure 3 summarizes the effect sizes for children’s gender-related preferences for toy vehicles and dolls, and the effect sizes for children’s gender-related preferences for broader groupings of boy-related and girl-related toys. The gender difference in preference for vehicles was significantly larger than the gender difference in preference for all boy-related toys, *t*(134) = 3.21, *p* = .002. Similarly, the gender difference in preference for dolls was significantly larger than the gender difference in preference for all girl-related toys, *t*(135) = 6.72, *p* < .001. Girls’ gender-specific preference for dolls was significantly larger than their gender-specific preference for all girl-related toys, *t*(129) = 5.61, *p* < .001. Boys’ gender-specific preference for vehicles was larger than their gender-specific preference for all boy-related toys, but this was not statistically significant, *t*(129) = 1.46, *p* = .148.

### Moderator Analyses

We found some covariance of measurement methods with child age, but not complete confounding, *F*(3,105) = 12.55, *p* < .001. Visual preference studies focused on infants, and children in these studies were younger than those in the studies using free play (*t*[61.78] = 7.76, *p* < .001), forced choice (*t*[32.96] = 8.23, *p *< .001), or naturalistic (*t*[6.96] = 5.46, *p* < .001) methods. We therefore conducted separate meta-regressions for each predictor, because one of the assumptions of meta-regression is that the predictor variables are independent. To test our assumptions, we conducted meta-regressions including interaction terms for the independent effects of age within each measurement method and using curvilinear terms for child age. All of the interaction and curvilinear terms were small and not statistically significant, so we proceeded with separate linear meta-regressions for method of measuring preference, age, and publication year.

### Method of Measuring Preference

**Fig. 4:**
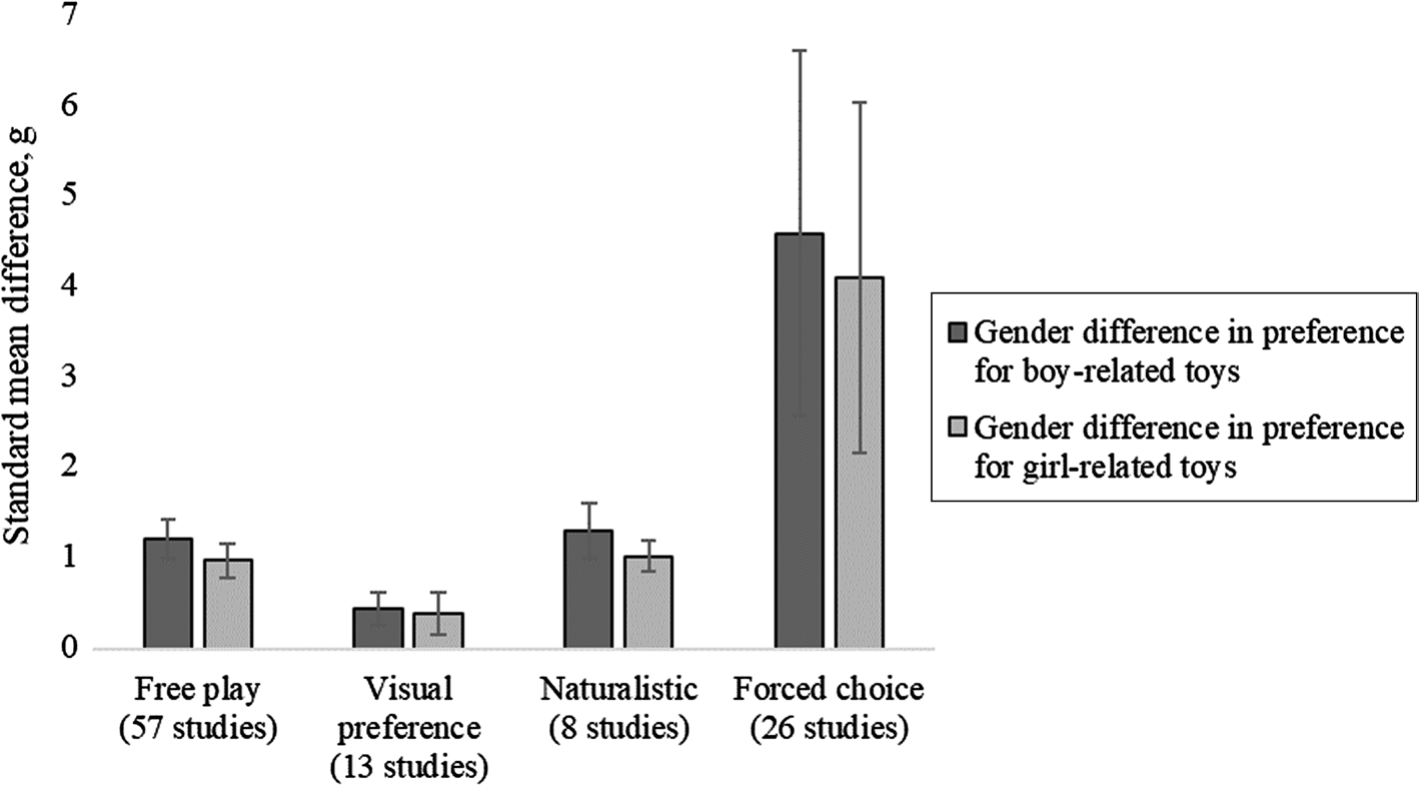
Standard effect sizes for free play, visual preference, forced choice, and naturalistic methods of measuring gender-related toy preferences. *Note*: neutral toys are not presented because almost all studies that gave children a neutral option were free play studies (22 of 29)

Method of measuring preference was operationalized as a categorical predictor with four levels: free play, visual preference, forced choice, and naturalistic methods. This four-level predictor was converted into a reference category (free play, since this was the largest category) and three dummy variables for the three other categories (visual preference, forced choice, and naturalistic). Analyses were multilevel meta-regressions with the gender effects (gender differences and gender-specific preferences) as the outcomes and dummy variables for different methods of measuring preference as the predictors. Figure 4 shows the standardized effect sizes for different methods of measuring gender-related toy preferences.

#### The Effect of Method of Measuring Preference on the Gender Difference in Preference for Boy-Related Toys

Forced choice methods showed larger gender differences in preference for boy-related toys than the reference category (free play methods), *b* = 3.05, 95% CI = 1.09–5.01, *p* = .002, but there were no significant differences between the reference category (free play methods) and visual preference, *b* = − 0.83, 95% CI = − 3.49 to 1.83, *p* = .542, or naturalistic methods, *b* = 0.18, 95% CI = − 2.68 to 3.04, *p* = .901.

#### The Effect of Method of Measuring Preference on the Gender Difference in Preference for Girl-Related Toys

Forced choice methods found larger gender differences in preference for girl-related toys than the reference category (free play), *b* = 2.70, 95% CI = 0.80–4.59, *p* = .005, but there were no significant differences between the reference category (free play) and visual preference, *b* = − 0.53, 95% CI = − 3.14 to 2.08, *p* = .689, or naturalistic methods, *b* = 0.06, 95% CI = − 2.72 to 2.84, *p* = .965.

#### The Effect of Method of Measuring Preference on Boys’ Gender-Specific Preference for Boy-Related Toys Over Girl-Related Toys

In boys, there was no significant effect of method (forced choice *b* = 2.09, 95% CI = − 3.42 to 7.60, *p* = .458, visual preference *b* = − 3.25, 95% CI = − 11.66 to 5.17, *p* = .450, naturalistic methods *b* = − 2.32, 95% CI = − 10.92 to 6.28, *p* = .597, compared to the reference category free play) on gender-specific preference.

#### The Effect of Method of Measuring Preference on Girls’ Gender-Specific Preference for Girl-Related Toys Over Boy-Related Toys

In girls, forced choice methods found larger gender-specific preference than the reference category (free play), *b* = 1.55, 95% CI = 0.17–2.93, *p* = .028, but there were no significant differences between the reference category (free play) and studies that used visual preference, *b* = − 0.40, 95% CI = − 2.42 to 1.63, *p* = .700, or naturalistic methods, *b* = 0.33, 95% CI = − 1.86 to 2.51, *p* = .770.

#### The Effect of Method of Measuring Preference on the Gender Difference in Preference for Neutral Toys

There was no significant effect of method of measuring preference (forced choice *b* = 0.12, 95% CI = − 0.77 to 1.02, *p* = .789, naturalistic *b* = − 0.35, 95% CI = − 1.31 to 0.61, *p* = .470, compared to the reference category, free play), on the size of the gender difference in children’s preference for neutral toys. These results could be unreliable, however, as 22 of 29 studies that provided a neutral toy option were free play studies. No studies used visual preference to measure gender differences in preference for neutral toys.

### Child Age

Age was operationalized as a continuous moderator, with each effect estimate assigned the average age reported for children in that sample (since individual-level data were not available).

#### The Effect of Age on the Gender Difference in Preference for Boy-Related Toys

The size of the gender difference in preference for boy-related toys increased significantly with child age, *b* = 0.02, 95% CI = 0.00–0.05, *p* = .027.

#### The Effect of Age on the Gender Difference in Preference for Girl-Related Toys

The size of the gender difference in preference for girl-related toys increased significantly with child age, *b* = 0.02, 95% CI = 0.00–0.05, *p* = .028.

#### The Effect of Age on Boys’ Gender-Specific Preference for Boy-Related Toys Over Girl-Related Toys

The size of boys’ gender-specific preference for boy-related over girl-related toys increased significantly with child age, *b* = 0.06, 95% CI = 0.02–0.11, *p* = .004.

#### The Effect of Age on Girls’ Gender-Specific Preference for Girl-Related Toys Over Boy-Related Toys

The size of girls’ gender-specific preference for girl-related over boy-related toys did not change significantly with child age, *b* = 0.01, 95% CI = − 0.01 to 0.03, *p* = .198.

#### The Effect of Age on the Gender Difference in Preference for Neutral Toys

The size of the gender difference in preference for neutral toys decreased significantly with child age, *b* = − 0.01, 95% CI = − 0.02 to − 0.01, *p* < .001.

### Publication Year

Publication year was operationalized as a continuous moderator, with each effect estimate assigned the year of publication of the study in which it was reported.

#### The Effect of Publication Year on the Gender Difference in Preference for Boy-Related Toys

There was no significant effect of publication year on the size of the gender difference in preference for boy-related toys, *b* < − 0.03, 95% CI = − 0.09 to 0.03, *p* = .309.

#### The Effect of Publication Year on the Gender Difference in Preference for Girl-Related Toys

There was no significant effect of publication year on the size of the gender difference in preference for girl-related toys, *b* < − 0.05, 95% CI = − 0.11 to 0.01, *p* = .103.

#### The Effect of Publication Year on Boys’ Gender-Specific Preference for Boy-Related Toys Over Girl-Related Toys

There was no significant effect of publication year on the size of boys’ preference for boy-related over girl-related toys, *b* = 0.02, 95% CI = − 0.15 to 0.19, *p* = .833.

#### The Effect of Publication Year on Girls’ Gender-Specific Preference for Girl-Related Toys Over Boy-Related Toys

There was no significant effect of publication year on the size of girls’ preference for girls’ toys over boys’ toys, *b* = − 0.03, 95% CI = − 0.08 to 0.01, *p* = .144.

#### The Effect of Publication Year on the Gender Difference in Preference for Neutral Toys

There was no significant effect of publication year on the size of the gender differences in preference for neutral toys, *b* = 0.01, 95% CI = − 0.00 to 0.03, *p* = .117.

## Discussion

We found a broad consistency of results across the large body of research on children’s gender-related toy preferences: children showed large and reliable preferences for toys that were related to their own gender. Thus, according to our review, gender-related toy preferences may be considered a well-established finding. Our results, with 75 studies and a range of toy preference measurements, complement and extend a previous meta-analysis of 16 studies focused on free play (Todd et al., 2018).

However, our meta-analyses also revealed some gaps that could prevent confident inferences about the drivers and consequences of children’s gender-related toy preferences. These gaps could form priority targets for future research. Our analyses also revealed some emergent patterns in the data, especially in how gender-related preferences for broad groups of toys differed in some respects from those for dolls and vehicles, how study results varied according to study method, and how gender-related differences in toy preferences related to child age.

### Toy Selection and Gender Categorization

The way that toys are selected, and categorized, as boy-related or girl-related, is not standardized in the present research. Studies in our review appeared to treat the gender categorization of toys as uncontroversial, even though, according to our review, it was not uncommon for toys to be assigned to different gender categories in different studies. For example, in some studies, blocks were classified as boy-related toys (e.g., Alexander & Saenz, 2012; Benenson et al., 1997; Fagot & Patterson, 1969), and in other studies they were classified as neutral toys (e.g., Cherney et al., 2003; Guinn, 1984; Wood, Desmarais, & Gugula, 2002). Similarly, drawing toys were sometimes categorized as girl-related toys (e.g., Berenbaum & Hines, 1992; Martin et al., 2013), and sometimes as neutral toys (e.g., Berenbaum & Snyder, 1995; Pasterski et al., 2005); and stuffed toys were equally likely to be classified as girl-related toys (e.g., DeLucia, 1963; Jacklin et al., 1973) as neutral toys (e.g., Alexander & Saenz, 2012; Idle et al., 1993; Moller & Serbin, 1996), but were also sometimes classified as boy-related toys (e.g., Stagnitti, Rodger, & Clarke, 1997). This pattern suggests that researchers sometimes disagree on what toys are boy-related, girl-related, or neutral.

In addition to finding that researchers sometimes disagreed on toy classifications, we also found that researchers typically did not report how they had selected toys for study or how they had assigned the toys to gender categories. We suspect that, in most cases, researchers used a simple heuristic method based on perceived cultural stereotypes. There are two problems with this type of approach. First, as noted above, toys categorized using this approach do not always fall into the same gender category in different studies. If one study includes a stuffed toy in the category “girls’ toys” and another study includes a stuffed toy in the category “neutral toys,” they may well report different results, even if the true underlying effect they are measuring is the same. Second, at its extreme, this problem may manifest as criterion contamination, in which gender-typed toys are defined by the results of the study. That is, the researchers may use many toys and select as “gender-related” toys the ones that they find to be differentially preferred by gender. At best, this tautology limits the generalizability of study results to other samples. At worst, it could invalidate the study.

Using methods that avoid confusion about toy categorization could be a priority for future research on children’s gender-related toy preferences. As also suggested by Fine (2015), this field could benefit from researchers specifying more clearly the ways in which they selected and categorized toys. Depending on the goal of the study, this selection and categorization might be based on different criteria. For example, a study examining whether stereotypes about children’s toy preferences relate to children’s actual preferences, might select toys based on adults’ independent ratings of the gender stereotyping of toys. In contrast, a study of the effect of a particular mechanism, such as social, cognitive, or hormonal influences, on toy preferences might select toys based on prior studies’ findings that certain toys are on average preferred by girls or boys. Overall, the important point is that researchers report more clearly how they selected toys and assigned toys to gender categories.

Researchers also have begun to investigate specific hypotheses about what characteristics of different toys might make them appeal more to boys or to girls. For instance, it has been suggested that color or shape might influence children’s gender-related preferences (e.g., Jadva et al., 2010; Weisgram et al., 2014; Wong & Hines, 2015). Similarly, it has been suggested that affordance of activity, motion, or propulsion might influence these preferences (Alexander & Hines, 2002; Benenson et al., 1997; Hassett et al., 2008; for a review, see Zosuls & Ruble, 2018). To evaluate these suggestions, it would be useful if researchers could provide color images, or full descriptions, of the toys used in the research they report. Similarly, it would be useful for this purpose, as well as for future reviews, if researchers could provide descriptive statistics, including means and SD or similar, by sex, for individual toys used, and not just for toy groupings.

To test whether the meta-analysis results were affected by researchers’ definitions of toy gender, we analyzed the subset of effect sizes that related to a very narrow definition of boy-related toys and girl-related toys: specifically, vehicles and dolls. These toys were the only ones for which sufficient data had been reported to allow reliable meta-analyses. The gender effects observed in the overall meta-analyses were broadly replicated with this more narrowly defined subset of toys, giving us confidence that our overall meta-analytic results were not entirely dependent on how researchers had chosen to categorize toys in regard to gender.

Furthermore, we found that girls’ gender-specific preference for dolls over vehicles was larger than their preference for broadly defined groups of girl-related toys. However, despite the large effect size, girls’ gender-specific preference for dolls over vehicles was not statistically significant, as this effect also showed large meta-analytic statistical variance. The large meta-analytic statistical variance is due to a combination of large variances in girls’ preference for dolls within the studies, variation between studies introducing additional statistical variance, and a smaller total number of studies that reported separate statistics for dolls as compared to broadly defined toy groups. In addition, the broadly defined toy groups included toys that, as mentioned above, were classified as neutral in some studies but girl-related in others. If toys are classified consistently, girls may show gender-related preferences at least as large as those of boys.

### Culture and Gender-Related Toy Preferences

Cultural perceptions of play, including play with toys, may differ in different cultural, ethnic, or socioeconomic groups. For example, play is viewed as central to children’s cognitive and social development in many Western, technologically developed societies, but as less important in more traditional societies (Roopnarine, 2010). Children in different cultures may also have different referential concepts for appropriate gender-related behavior, due to cultural variation in gender norms (Pfeiffer & Butz, 2005; Wood & Eagly, 2002). This possibility is particularly relevant to toy preferences, because there may be cultural variations in the toys that are available, culturally relevant, and gender-related.

Nevertheless, little empirical research is presently available on cultural variation in gender-related toy preferences. Our review revealed that most toy preference studies focus on the U.S., Canada, the UK, and Australia. Of those studies conducted outside English-speaking industrialized nations, one was conducted in France (Le Maner-Idrissi, 1996), one in Finland (Lamminmäki et al., 2012), four in Sweden (Nelson, 2005; Nordenström, Servin, Bohlin, Larsson, & Wedell, 2002; Serbin et al., 2001; Servin, Bohlin, & Berlin, 1999), and one in the Netherlands (van de Beek et al., 2009). An additional study included some participants from Hungary, along with participants from the UK (Turner & Gervai, 1995). These studies did not report different results to the studies from the English-speaking countries, even when researchers had specifically hypothesized that they would (e.g., Nelson, 2005). In global perspective, however, these countries are very similar in terms of industrialization, wealth, education, media access, democracy, and gender equality. Consequently, children in these countries probably have very similar toys available to them and similar access to information about dominant social stereotypes around these toys. It remains an open question, then, whether children in cultures with radically different stereotype referents and social norms would show the same gender-related toy preferences to those found in the current meta-analysis.

We did not formally investigate other aspects of cultural diversity, such as ethnicity and socioeconomic status, because these also have not received much attention in empirical studies of gender-related toy preferences. Participants in most toy preference studies are not very ethnically diverse, and so it may not be practical to report results by ethnicity. We found three studies (out of our total 75) that reported toy preferences by ethnicity. Two of these studies were conducted in the USA and reported no significant differences in gender-related toy preferences between children of Hispanic and non-Hispanic background (Goble, 2012), or Native American and non-Native American background (Guinn, 1984). In contrast, another study based in the U.S. found that ethnicity might affect children’s preferences for gender-related activities, including play with toys, via children’s social networks (Martin et al., 2013). Furthermore, in recent years, the wider field of gender development research has paid increasing attention to the intersectionality of gender, ethnicity, and other identities (e.g., Shields, 2008). This trend in the wider field may translate in future to more studies investigating gender-related toy preferences in diverse social groups.

### Methods of Measuring Toy Preference Are Important

Studies may find different gender effects on children’s toy preferences, depending on the method they use to measure toy preferences. We evaluated four categories of study methods: free play methods, where children were given access to a set of toys and observed playing, however, they liked; visual preference measures, where children were asked to look at pictures of toys; forced choice methods, where children were asked to choose toys or pictures of toys, typically in front of an experimenter; and naturalistic methods, where children’s toy options were not predefined by the researchers or other adults. We found that forced choice methods consistently showed larger gender differences than other methods.

There are two possible explanations for this pattern. One is the potential demand characteristics of forced choice paradigms. A request to publicly choose an option may be interpreted as evaluative by children, who then feel obliged to give the answer that they feel is “correct,” rather than indicate their actual preference. Children’s propensity to misunderstand requests for information as tests has been noted in other contexts (e.g., Lamb et al., 2003). Another possibility is that the paradigm creates a false dichotomy. In forced choice methods, the child is usually presented with one boy-related option and one girl-related option and asked to choose between them. There is usually not a neutral option, and, generally, the child must choose only one option and reject the other. In contrast, in a free play paradigm, children typically have more response options available, such as several toys associated with each gender, or neutral toys as well as gender-related toys. Even if only two toys are available, the child has more options than in most forced choice paradigms. For example, if a doll and a car are available, a child may choose to play with the doll, play with the car, play with both the doll and the car, or play with neither. In most forced choice methods, however, children must choose one and only one of two options.

Forced choice methods, in their current form, do not give comparable results to other methods of measuring gender-related toy preferences. Nevertheless, forced choice methods can be an efficient and easily administered measurement tool and therefore may be appropriate for studies where, for example, data need to be collected across a very large group or under difficult conditions. Future investigators wishing to measure gender-related toy preferences with an easily administered tool might do so, however, with the aim of minimizing artificial inflation in effect sizes. For instance, a procedure in which the experimenter cannot see which option the child selects, and the child knows that their response is not seen, might be useful. It also might be useful to include neutral options, as well as gender-related options, and allow the range of possible choices to include “both” or “neither.” These modifications of forced choice methods could provide results that are more comparable to other methods of measuring toy preference and perhaps are more reflective of children’s actual gender-related preferences.

### Child Age and Gender-Related Toy Preferences

We found that gender differences in preferences for gender-related toys increased linearly with child age. Our results further suggested that this pattern could be explained by boys’ showing increased preference for gender-typical over gender-atypical toys with age, while girls’ preferences for gender-typical over gender-atypical toys did not increase significantly with age. Similarly, the previous meta-analysis of free play studies (Todd et al., 2018) found an increase in gender-related play with age in boys, although they did not find increasing gender differences. This may reflect a difference in the power of the two meta-analyses; the previous meta-analysis included 16 studies, whereas the current meta-analysis included 75 studies. We did not find significant curvilinear effects of age on children’s gender-related toy preferences.

Our findings of linear effects contrast with those of some prior investigations of age effects on children’s gender-related toy preferences. For example, Campbell et al. (2000) measured infants’ gender-related visual preferences longitudinally at ages 3, 9, and 18 months. They found that preferences did not change with age, but the infants were all very young compared to the age range in the wider literature and in the current meta-analysis.

In contrast, our meta-analytic findings suggest that boys’ and girls’ gender-related toy preferences increase with age in a linear fashion. These findings resemble findings for a broader measure of children’s gender-typical behavior, the Pre-School Activities Inventory (PSAI). The PSAI is a 24-item parent report inventory that asks about children’s gender-typed toy preferences and about children’s gender-related activity and playmate preferences. A longitudinal, population study in which the PSAI was completed by a parent to describe their child at ages 2, 3, and 5 years also found that both boys and girls became increasingly gender-typed with age (Golombok et al., 2008).

Our results suggest that children’s toy preferences might become more gender-related with age, as predicted by several theories of gender development. Children might be encouraged, through socialization pressures such as modeling and reinforcement, to prefer same gender-related toys, and the effects of this socialization may accumulate as they get older (Fagot, Rodgers, & Leinbach, 2000). Additionally, based on their early gender-related toy interests, children might gravitate to different social environments, enhancing their early preferences (Liben & Bigler, 2002; Martin et al., 2013). Finally, differences in children’s prenatal and early postnatal hormone exposure may dynamically interact with social environments and cognitive processes to increase children’s gender-related preferences over time (Hines, 2012). Together, these social and cognitive effects, and their interactions with early hormonal influences, may explain the linear increase in gender-related differences with age.

The findings of our meta-analysis, however, are not a substitute for a large, longitudinal study of children’s gender-related toy preferences. We used meta-analytic techniques to compare gender-related preferences in children from different age groups, reported in different studies. Our analysis, therefore, was cross-sectional and does not have the inferential power of a well-controlled longitudinal study. Our results would be best confirmed by a future longitudinal study of children’s gender-related toy preferences from infancy to pre-pubertal age. The longitudinal parent report study using the PSAI (Golombok et al., 2008) is the closest existing example and found similar results to our meta-analysis.

### Gender-Related Toy Preferences Over Time

We found no change in the magnitude of gender-related differences in toy preferences across year of publication. The results of the moderator analyses suggested that gender effects on children’s toy preferences have remained generally constant in magnitude across the past five decades. This finding might seem surprising. Since the earliest studies on gender-related toy preferences, gender-atypical behavior and preferences have become increasingly socially acceptable. Perhaps the lack of any discernible pattern of change results from different social pressures influencing gender-related toy preferences in different directions. For example, growing acceptance of gender-atypical behavior may be countered by increasing gender segregation of the toy market.

Contrary to our results, a previous meta-analysis of children’s toy preferences (Todd et al., 2018) found that boys and girls played more with gender-related toys in earlier studies than in more recent studies. Todd et al. suggested that increasing gender equality in Western societies could influence children to play with neutral toys, due to increased advertising to children about gender-neutral toys. A recent analysis of online toy marketing, however, found that more toys were marketed for “boys only” or for “girls only” than for both (Auster & Mansbach, 2012), and an analysis of department store catalogs concluded that gender differentiation in toy advertising had increased since the 1980s as marketers employed gender stereotypes to encourage sales (Sweet, 2013). Taken together, these analyses challenge the view that gender-related toy advertising is decreasing with time. Alternatively, the previous finding could be partly explained by the smaller time frame considered in the prior meta-analytic review; the prior review covered about 35 years of research, while the present review covered 50 years.

It may be that children’s preferences are robust to social influences at this macrolevel; or that, despite social change, the underlying cultural environment regarding gendered toys has not changed. A similar result was found in a systematic review of gender stereotypes from the 1970s to the present. Rudman and Glick (2008) hypothesized that women’s changing social roles would be reflected in changing stereotypes of women. Although they found a change in women’s self-concept over time, they also found that more general stereotypes of women’s personalities had not changed. They suggested that the lack of change might be due to people viewing personality as part of the fundamental essence of gender, and therefore being reluctant to modify their stereotypic beliefs about personality. A similar explanation may also apply to toy preferences: if people view toy preferences as an essential part of a child’s gender, they may be unlikely to change their gender-related beliefs about toy preferences. Children may then adapt their actual toy preferences to reflect broader societal beliefs.

### Limitations

The meta-analysis could only include data that were reported in the individual toy preference studies. Therefore, we could not analyze variables such as color or shape, or individual toys other than dolls and vehicles. In future research, if investigators report more information about toy characteristics and about individual toys, it may be possible to discover more about what characteristics of different toys make them more likely to be preferred by one gender or another.

Our literature search covers papers published to March 2014 and does not include papers published outside of this time frame. More recent papers may therefore be missing from the current meta-analysis. The current meta-analysis, however, synthesizes 50 years of research on toy preferences and finds that toy preference effect sizes have not changed significantly over time. Thus, results from a new review including more recent papers would be unlikely to differ from what we report.

We focused on gender-related preferences in typically developing children. Some studies selected participants specifically because they were not typically developing (for example, clinical samples of children with genetic variants causing atypical early hormone environments, or children who showed gender-related behavior that was noticeably different from their peers). To include these atypical populations in our study might have skewed the results, so we did not include them. Our results, therefore, may not apply to clinical populations.

Additionally, we meta-analyzed only direct measures of children’s toy preferences. We did not, for example, include parent report measures. Similarly, we did not include broader aspects of children’s gender-related behavior, such as activity preferences, playmate preferences, or sex role identification (e.g., Brown, 1956). Additionally, we did not search for these broader terms, so we may have missed papers that included toy preferences in a broader measure of sex role identification or androgyny (e.g., Zucker & Torkos, 1989). It would be interesting to know whether meta-analyses from these other sources of data and types of gender-related behavior would show similar outcomes. We hope that the current systematic review and meta-analysis will encourage such studies.

### Conclusions

Meta-analyses of gender-related differences in children’s toy preferences found that gender differences and gender-specific effects on children’s toy preferences are large and reliable, and that some toys that researchers have classified as neutral may actually be preferred by girls. Also, the meta-analytic results suggest that girls and boys show gender-related differences of similar magnitude, both for broad groups of toys and for dolls and vehicles, specifically. In addition, forced choice methods show larger gender-related differences than other methods, and gender-related differences increase with age, but have not changed in size over historical time. Few prior studies have reported data for individual toys or for varied cultures, ethnicities, or socioeconomic groups. Future research could usefully report how toys were chosen for study and classified into gender categories and report descriptive statistics for the individual toys used. Useful future studies might analyze children’s gender-related toy preferences in different cultures, ethnicities, and socioeconomic groups.

## Footnotes

1 Interested readers may contact corresponding author for the references that were gathered in the systematic search but were not included in the meta-analysis.
